# Recent Advances in Aptamer Discovery and Applications

**DOI:** 10.3390/molecules24050941

**Published:** 2019-03-07

**Authors:** Yang Zhang, Bo Shiun Lai, Mario Juhas

**Affiliations:** 1 College of Science, Harbin Institute of Technology, Shenzhen 518055, China; 2 School of Medicine, Johns Hopkins University, Baltimore, MD 21205, USA; boshiun@jhmi.edu; 3 Institute of Medical Microbiology, University of Zurich, Gloriastrasse 28/30, CH-8006 Zurich, Switzerland; mjuhas@imm.uzh.ch

**Keywords:** aptamer, systematic evolution of ligands by exponential enrichment (SELEX), diagnostics, therapeutics, biosensor, nanorocket

## Abstract

Aptamers are short, single-stranded DNA, RNA, or synthetic XNA molecules that can be developed with high affinity and specificity to interact with any desired targets. They have been widely used in facilitating discoveries in basic research, ensuring food safety and monitoring the environment. Furthermore, aptamers play promising roles as clinical diagnostics and therapeutic agents. This review provides update on the recent advances in this rapidly progressing field of research with particular emphasis on generation of aptamers and their applications in biosensing, biotechnology and medicine. The limitations and future directions of aptamers in target specific delivery and real-time detection are also discussed.

## 1. Introduction

Target-specific therapy was discovered by the inventor of chemotherapy, Paul Ehrlich [1]. The ensuing development of hybridoma technology for generating monoclonal antibodies, which realized Ehrlich’s vision [2], along with the therapeutic success of antibodies led to the development of a number of novel antibody drugs. The examples of these drugs include trastuzumab targeting receptor tyrosine-protein kinase 2 ERBB2 implicated in ovarian and breast tumors [3], anti-CD20 chimeric mAb rituximab developed for treating B-cell non-Hodgkin lymphoma [4], and Vedolizumab inhibiting integrin α4β7, which plays an important role in ulcerative colitis and Crohn’s disease [5].

The idea to use nucleic acids to recognize protein targets only emerged later as a result of HIV research. It was shown that the trans-acting response element (TAR)-containing RNA sequences can inhibit HIV replication with high affinity and specificity [6].

Aptamers are short, single-stranded oligonucleotides (DNA or RNA) that bind to targets with high affinity and specificity by folding into tertiary structures [7,8]. Aptamers have been extensively used in basic research, to ensure food safety and to monitor the environment. Furthermore, aptamers have promising role in clinical diagnostics and as therapeutic agents. Although aptamers recognize and bind targets of interest just like antibodies, they have a number of advantages, such as shorter generation time, lower costs of manufacturing, no batch-to-batch variability, higher modifiability, better thermal stability and higher target potential ranging from ions to live animals (Table 1). A more thorough comparison of aptamers and antibodies was reviewed by Zhou and Rossi [9]. Our review provides an update on recent advances in the field of aptamers with particular focus on the methods of generating novel aptamers and the applications of aptamers in biosensing, biotechnology and biomedicine.

## 2. Generation of Aptamers

A number of approaches were developed in recent years to generate aptamers more reliably and efficiently. These include the systematic evolution of ligands by exponential Enrichment (SELEX) approach and its variations, such as immunoprecipitation-coupled SELEX (IP-SELEX), capture-SELEX, cell-SELEX, capillary electrophoresis-SELEX (CE-SELEX), atomic force microscopy-SELEX (AFM-SELEX), and artificially expanded genetic information system-SELEX (AEGIS-SELEX), which are herein reviewed. The key aspects, advantages, and disadvantages of each SELEX method [9,10,11,12] are highlighted in Table 2.

### 2.1. Systematic Evolution of Ligands by EXponential Enrichment (SELEX)

**Figure 1:**
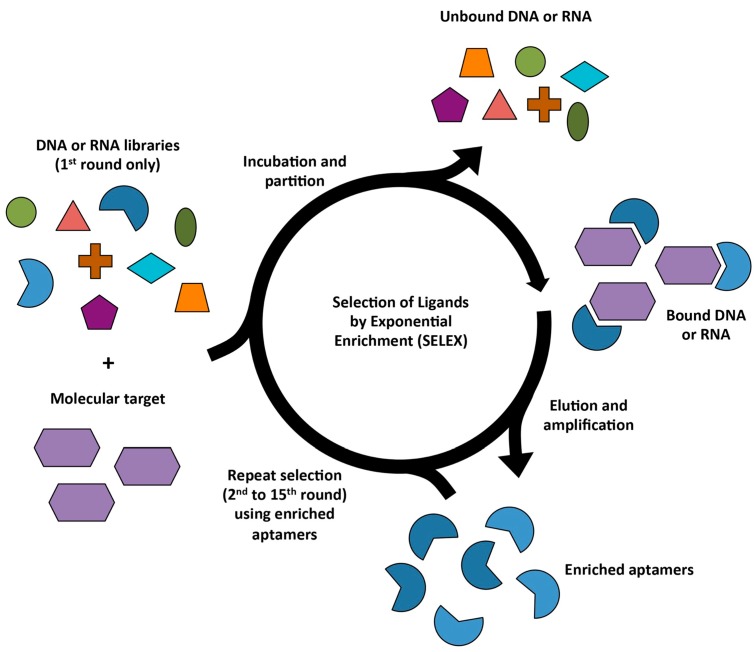
Schematic representation of SELEX (Systematic Evolution of Ligands by EXponential Enrichment). The starting single-stranded DNA or RNA library (10^14^~10^16^ random oligonucleotides) is composed of sequences 20~100 nucleotides in length with a random region in the middle flanked by fixed primer sequences. After incubation with the target of interest, the bound oligonucleotides are partitioned from unbound sequences and amplified by PCR. The resulting enriched DNA pool is used for the next round of selection.

SELEX is a standard method of generating aptamers, where the target of interest is first incubated with a pool of 10^14^~10^16^ single-stranded random oligonucleotides. Oligonucleotides in the SELEX library typically consist of 40~100 nucleotides, which harbor a random region in the middle and fixed sequences on both ends. Subsequently, the target-binding oligonucleotides are separated from the unbound ones. The bound DNA oligomers are then eluted and amplified by PCR. After several rounds of selection, the resulting DNA sequences (aptamers) with high affinity and specificity are enriched in the pool and sequenced (Figure 1) [7,8]. The most common modifications toward traditional SELEX method aimed to simplify the SELEX procedure or to generate improved aptamers include changing binding conditions, platform on which the selection is performed, type of target, library design and immobilization matrix. Besides DNA libraries, RNA libraries have also been successfully used for SELEX [9,13,14]. The differences in the majority of RNA SELEX protocols compared to DNA SELEX include the requirement of the protection of RNA from RNAases, amplification by T7 RNA polymerase and reverse transcription step before PCR. Consequently, the 5′-primer used for RNA SELEX usually encodes a promoter for T7 RNA polymerase [13].

### 2.2. Immunoprecipitation-Coupled SELEX (IP-SELEX)

The affinity and specificity of aptamers largely depend on their targets’ three-dimensional structures. As the three-dimensional targets of artificial, recombinant proteins can differ from their native conformation, it is suggested that the in vitro selection of aptamers might not be the best approach to select aptamers with the highest affinity and specificity under the standard physiological conditions [15]. IP-SELEX was developed to address this issue. Although IP-SELEX is comparable to the traditional SELEX method, it includes immunoprecipitation step to pull down the desirable targets in their native form. This leads to the enrichment for aptamers that retain the ability to recognize proteins under the normal physiological conditions. In the first round of IP-SELEX selection, cells harboring the protein of interest are incubated with ssDNA library in the suitable buffer and condition. Subsequently, cells are lysed, and the target protein is immunoprecipitated using antibody-coated beads. Following several rounds of washing, the protein-aptamer complexes are eluted from the beads and DNA sequences are PCR amplified for the next round of selection. The process of selection for the oligonucleotides with the high specificity and affinity against a desired target is repeated, thus leading to their final enrichment in the aptamer pool. The selected aptamers are then identified by sequencing [16]. IP-SELEX has been successfully used for the identification of anti-CD8 aptamers [17,18].

### 2.3. Capture-SELEX

In comparison to most SELEX methods, which require immobilization of targets on a solid surface, Capture-SELEX is used for a soluble small molecule targets. Capture-SELEX utilizes a special DNA library harboring fixed domain in the center flanked by two random domains, each of which is flanked by primer-binding sequences. As the fixed domain in the center is designed to be complementary to a biotinylated antisense oligonucleotide, this allows immobilization of the DNA library onto avidin-coated beads by hybridization. Aptamers that are able to form complexes with their targets detach from beads and solubilize, which allows their collection and PCR amplification [19]. Capture-SELEX strategy has been used successfully to select both DNA and RNA aptamers against small organic molecules in solution [20]. Furthermore, Capture-SELEX has been used recently to select aptamers against furaneol [21], penicillin [22], fluoroquinolone antibiotics [23], tobramycin, and other aminoglycoside antibiotics [24,25].

### 2.4. Cell-SELEX

In Cell-SELEX, live cells are used to select aptamers [26]. Cell-SELEX is considered to have practical applications particularly in oncology, as this approach can select aptamers specific for the cancer cell targets. Employing Cell-SELEX, aptamer-based probes have been developed for a number of cancers, including cervical cancer [27,28], ovarian cancer [29,30], liver cancer [31], prostate cancer [32], breast cancer [33,34], glioma, [35], colorectal carcinoma [36], and lung carcinoma [37]. A number of cell-SELEX variants have been developed in the last years. These include cell-internalization SELEX where aptamers are transported intracellularly and internalized by the analyzed cells, 3D cell-SELEX which involves development of 3D laboratory cell cultures to develop aptamers and cross-over SELEX which utilizes both the cells and the purified protein to increase the selection efficiency [38,39] (Table 2). Ligand-guided selection (LIGS) is another interesting variant of Cell-SELEX which selects aptamers specifically binding known cell surface proteins [11]. LIGS is therefore particularly useful for the generation of aptamers with high specificity and has been utilized also for the selection of aptamers with high specificity against membrane-bound immunoglobulin M (mIgM) [40,41,42]. Furthermore, flow cytometry has been successfully integrated into the Cell-SELEX approach to isolate aptamer-bound cells and eliminate dead cells [15,43]. The main disadvantage of Cell-SELEX and its variants is that the whole process is time consuming and requires a certain level of technical expertise [11] (Table 2). The main advantages of cell-SELEX and its variants are the abundance of molecules on the cell surface with the potential to become targets and their native conformation which is important for diagnostic and therapeutic applications [31,44]. Furthermore, as the whole cells are applied to the selection, there is no need to have the prior knowledge of the biomarkers. In cell-SELEX, the successful selection leads to the generation of aptamers against unknown biomarkers which can be subsequently identified by purification and analysis of the aptamers [11] (Table 2).

### 2.5. Capillary Electrophoresis-SELEX (CE-SELEX)

CE-SELEX exploits capillary electrophoresis, an analytical technique for separating ions based on their electrophoretic mobility, to generate aptamers with high affinity and specificity in as few as one to four rounds of selection compared to more than 15 selection rounds required for traditional SELEX [12,45]. Capillary electrophoresis in CE-SELEX separates bound DNA molecules from unbound ones in a solution, thus eliminating linker and kinetic bias by stationary support preparation and washing. CE-SELEX was first used to generate aptamers against neuropeptide Y and IgE [46,47]. A number of aptamers have been successfully generated employing CE-SELEX, including those against alpha-fetoprotein and activated protein C [48,49]. Furthermore, a fraction collection approach has been integrated into CE-SELEX for the partition of a bound DNA–target complex in order to generate the target specific binding aptamers in a single round of selection [50]. Recently, CE-SELEX has been successfully used to generate aptamers against glypican-3, a tumor biomarker for the early diagnosis of hepatocellular carcinoma [51]. However, capillary electrophoresis is restricted to the selection against high molecular weight targets. In addition the diversity of the libraries that are handled in CE-SELEX is largely reduced compared to SELEX (Table 2).

### 2.6. Microfluidic-SELEX (M-SELEX)

M-SELEX based on microfluidics system capable of handling small volumes of fluid was employed to obtain high affinity aptamers against diverse protein targets recently, including influenza A nucleoprotein (infA NP), ovarian cancer and cardiovascular biomarkers [52,53,54,55]. M-SELEX is a universal and automatable approach for rapid generation of aptamers with high affinity and specificity at the microscale level. The most notable improvements to M-SELEX include a micro-magnetic separation device to increase the efficiency of separation [56,57], sol-gel technique [58,59], bead-based acoustophoresis technique [60], and microarray-integrated microfluidic chip technique [12,61].

### 2.7. Atomic Force Microscopy-SELEX (AFM-SELEX)

AFM-SELEX exploits a high resolution atomic force microscopy (AFM) with cantilever as a probe to measure the weak force between the sample surface and the probe to create the three-dimensional images of the sample surface. AFM-SELEX approach utilizing AFM dynamic affinity force measurement has been developed recently to obtain aptamers with increased affinity [62]. Furthermore, AFM-SELEX has been also successfully applied to develop aptamers against human serum albumin recently [63]. In this study, aptamers against human serum albumin could be selected in the fourth round, based on the measuring of the DNA-duplex interactions by AFM [63].

### 2.8. Artificially Expanded Genetic Information System-SELEX (AEGIS-SELEX)

AEGIS-SELEX utilizes modified libraries with the artificially expanded genetic code. This includes incorporation of hydrophobic base 7-(2-thienyl) imidazo (4,5-b) pyridine (Ds) nucleotides into a random natural nucleotides sequence library to obtain aptamers with the increased affinity [64]. AEGIS-SELEX has also been used to incorporate four natural and two synthetic nucleotides (commonly referred to as P and Z) [65]. Rearranging donor and acceptor of hydrogen bond on nucleobases by incorporating unnatural base pairs was shown to significantly increase the functional diversity of aptamers. Furthermore, AEGIS-SELEX has been used successfully for the laboratory evolution of artificially expanded DNA to generate aptamers targeting toxic form of *Bacillus anthracis* protective antigen [66]. Current applications of AEGIS-SELEX are limited mainly by the poor recognition of the unnatural base by the naturally occurring DNA polymerases. However, this obstacle can be overcome by the direct evolution of polymerases able to recognize unnatural bases [67].

### 2.9. Animal-SELEX

**Figure 2:**
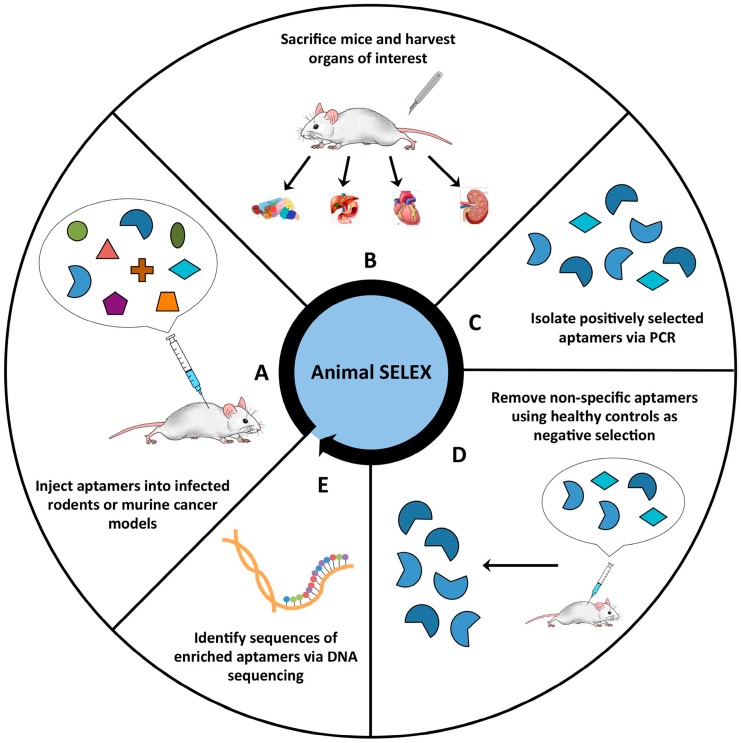
Flowchart of animal SELEX. Animal SELEX can be used to generate aptamers specific to target tissues. (**A**) Aptamer libraries are first injected into the target mice. (**B**) After inoculation, the organs of interest are harvested. (**C**) The selected aptamers are isolated and amplified by PCR. (**D**) After rounds of selection, counter selection can be performed by inoculating aptamer pool into the healthy mouse tissues. (**E**) The aptamer sequences with high affinity and specificity to the target tissues of interest are selected and identified by sequencing.

In the whole animal in vivo SELEX, mouse cancer models or pathogen-infected mice can serve as a positive target. Here, aptamer libraries are first injected into the target mice (Figure 2A) and, following inoculation, the organs of interest harvested (Figure 2B). Next, the selected aptamers are isolated and amplified by PCR (Figure 2C). After selection, counter selection can be introduced by inoculating the aptamer pool into the healthy mouse tissues (Figure 2D). The resulting sequences of the disease-specific aptamers with high affinity and specificity to target tissues can be enriched and identified by sequencing (Figure 2E). Aptamers penetrating the blood–brain barrier (BBB) were successfully developed using this selection strategy against brain tissue from mice [68].

Animal-SELEX was employed recently to identify bone targeting aptamer in a mouse model with prostate cancer bone metastasis [69], Toll-like receptor 4 (TLR4) blocking aptamers for use as acute stroke treatment [70], aptamers with the potential to be used as biomarkers for neurological disorders [71]. Furthermore, animal-SELEX in a murine model of lymphoma has been used recently to screen DNA aptamers with homing specificity to lymphoma bone marrow involvement [72].

## 3. Applications of Aptamers

Analogically to monoclonal antibodies, aptamers can specifically recognize and bind to their target [73]. Therefore, following their isolation, aptamers can be utilized for molecular recognition of their targets. Consequentially, aptamers have a number of diagnostic and therapeutic applications, such as biosensors and target inhibitors. Due to simple preparation, easy modification, and stability, aptamers have been used in the diverse areas within molecular biology, biotechnology, and biomedicine.

### 3.1. Aptamers as Diagnostics

The high affinity and specificity of aptamers make them ideal diagnostic agents with the potential to replace conventional antibodies in clinical diagnosis, environmental protection, and food safety. Like monoclonal antibodies, aptamers can be used for the molecular recognition of their respective targets. Aptamers have been successfully used for pathogen recognition, cancer recognition, monitoring environmental contamination, and as stem cell markers.

#### 3.1.1. Pathogen Recognition

The fluorescence resonance energy transfer (FRET)-aptamers were developed as a novel high-throughput screening tool against *Escherichia coli* outer membrane proteins to detect enterotoxaemia *E. coli* (ETEC) K88 [74]. Furthermore, aptamers were utilized to detect surface proteins of *Campylobacter jejuni* [75]. In addition to using purified bacterial proteins as targets, the whole bacterium-based SELEX procedure was applied to detect *E. coli* [76], *Lactobacillus acidophilus* [76], *Staphylococcus aureus* [77], the virulent strain of *Mycobacterium tuberculosis* [78], *Vibrio parahemolyticus* [79], *Shigella sonnei* [78], and *C. jejuni* [80]. This led to development of aptamers with increased affinity and specificity. SELEX-based approaches can be also used to generate molecular probes for detecting viral infections, such as vaccinia virus [81], herpes simplex virus [82], hepatitis C virus [83,84], hepatitis B virus [83,84], human immunodeficiency virus [85], influenza virus [86], and Severe Acute Respiratory Syndrome (SARS) coronavirus [87]. Furthermore, SELEX has been used successfully to generate aptamers for the detection of a number of parasites, such as *Trypanosoma* spp. [88], *Leishmania* spp. [89], *Plasmodium* spp. [90,91,92,93,94,95,96,97,98,99], *Cryptosporidium parvum* [100], *Entamoeba histolytica* [101]. A more thorough overview of the recent advances on aptamers as diagnostics of protozoan parasites was reviewed by Ospina-Villa et al. [73].

#### 3.1.2. Cancer Recognition

Development of aptamers for a reliable and timely cancer diagnosis and prognosis evaluation is of the highest importance. To address this issue, aptamers have been developed for the detection of a number of cancer-related biomarkers [102], including multiple tumor-related proteins in living cancer cells, such as MUC1 (mucin 1), HER2 (human epidermal growth factor receptor 2), and estrogen receptor [102]. Aptamers for the detection of the MCF-7 breast cancer cells [103] and leukemia CCRF-CEM cells were also developed recently [104]. In addition, aptamers have been successfully used for the detection of a number of tumor-related soluble biomarkers, including carcinoembryonic antigen (CEA), prostate specific antigen (PSA) [105,106]. Fluorescently labeled aptamers showed high detection rates of the metastatic tumor tissues [107]. Furthermore, aptamers were successfully used also for the in vivo imaging of lymphoma, adenocarcinoma, leukemia, glioblastoma and other cancer types [102,108]. The current developments in the application of aptamers for the molecular recognition of cancer have been summarized in a recent review by Sun et al. [109].

#### 3.1.3. Stem Cell Recognition

Progress in developing aptamers against stem cells has been slow. There are only a few aptamers against stem cells markers such as cancer stem cells (CSCs), including cancer cell surface biomarkers: Epithelial cell adhesion molecule (EpCAM), CD133, CD117, and CD44 [110], and those for mouse embryonic stem cells [111].

#### 3.1.4. Monitoring Environmental Contamination

Aptamers have the potential to monitor and to minimize the environmental pollutants and the resulting illnesses. Antibiotics, heavy metals, toxins, and pathogens can be toxic to nervous, endocrine, and reproduction systems. Antibiotics used for farm animals may accumulate in the animal tissues and transmit to human upon ingestion. To address this issue, aptamers have been developed against some antibiotics, such as chloramphenicol [112] and tetracycline [113].

Furthermore, aptamers for a number of environmental toxins have been developed recently. These include aptamers against ochratoxin A (OTA) [114], bacterial endotoxins [115], and bisphenol A [116]. Aptamers against mercury [117,118], arsenic [119,120,121], copper [122], and lead [123] have also been generated to identify heavy metal contamination.

Aptamers for the detection of various pesticides in the environment have been also developed recently, such as fungicide carbendazim [124], acetamiprid and atrazine [125,126], and chlorpyriphos [127].

In addition, aptamers have been generated against various types of herbicides [128] and insecticides [129], which may cause reproductive damage in humans. Abraham et al. reported on the identification and characterization of an ssDNA aptamer against herbicide atrazine, recently [130].

### 3.2. Aptamers Used in Biosensors

**Figure 3:**
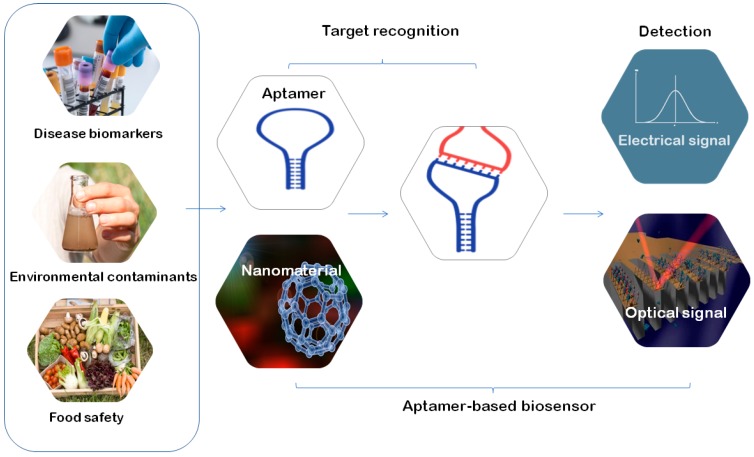
Aptamers used in biosensors. Aptamer-based biosensors are used to detect disease biomarkers, monitor environmental contaminants, or to ensure food safety. Aptamers can be further enhanced by different nanomaterials or biomaterials. The signal of detection in the most of the recently developed aptamer-based biosensors is based on electric or optic/fluorescent signal.

The ease of which different aptamer structures can be generated together with their ability to bind specific targets by forming stable tertiary structures has led to widespread applications of aptamers in biosensors [123,131,132,133] (Figure 3).

Besides monitoring environmental contamination described above, examples of the recently developed biosensors include the following: (1) Aptamer-based biosensors to target disease biomarkers, such as platelet-derived growth factor BB (PDGF-BB), a cancer related protein, to help early diagnosis and prognosis of cancer development [134]. (2) Biosensors for the azole class of antifungal drugs with application in the therapeutic drug monitoring of patients with invasive fungal infections [135]. (3) Biosensors harboring DNA aptamers targeting B-lactoglobulin for the detection of milk allergen [124]. (4) Highly sensitive biosensors for the detection of human epidermal growth factor receptor 2 with application in real-time detection of breast cancer cells [136]. (5) Biosensors for the detection of bisphenol A [137].

Biosensors can be enhanced by using different nanomaterials (Figure 3). A good example of this is the graphene-based biosensor targeting B-globulin for the detection of milk allergen [124] and the biosensor for the detection of human epidermal growth factor receptor 2 [136] described above. In the latter, ultrafine graphene nanomesh, a continuous 2D graphene nanostructure with a high density of holes punched in the basal plane, has been created to improve the signal on/off ratio [136]. Biosensors can be further enhanced by using biomaterials, such as antibodies to form a "sandwich" structure. In the biosensor developed by Wiedman et al. for the azole class of antifungal drugs, two aptamers were combined to form a "sandwich" structure [135].

Currently, change in the biosensor’s electric signal (current and resistance) upon binding to analytes is the major principle of detection, while the electrochemical impedance spectroscopy (EIS) and cyclic voltammetry (CV) are usually used to monitor aptamer–ligand complexes occurring on electrode surface [133]. DNA-based electrochemical biosensors have been widely used for the detection of heavy metal ions, including mercury, lead, and copper [138]. Fluorescence-labeled aptamer probes have also been widely used in biosensors (Figure 3). A novel label-free aptasensor for acetamiprid through fluorescence resonance energy transfer (FRET) involves the conformational change taking place after the aptamer binds to acetamiprid and its effect on the stability of the gold nanoparticles in solution. In this biosensor, dual-colored Au NCs (gold nanoclusters) excitable by single-wavelength excitation were used as energy donors to achieve simultaneous detection of multiple tumor markers [139]. Another recent example of the fluorescent biosensor is the biosensor for the detection of bisphenol A based on the adsorption ability of a nano-sized iron metal–organic framework (Fe-MIL-88B–NH2) to a DNA aptamer [137].

To further enhance the affinity of detection, recent studies have focused on three aspects: reconstructing aptamers, modifying electrodes and exploiting the inter-substance interactions. It has been demonstrated that removing the non-binding domain of aptamers could increase the binding affinity and specificity of the aptamer-target complex by improving the density of the binding domains [140]. Furthermore, the rolling circle amplification increases the length of the aptamer, thus increasing the number of binding domains. Electrochemical roughening approach also enhanced the signaling of electrochemical sensors by increasing the microscopic surface area of gold electrodes [141]. This simple method does not require pretreatment compared to the conventional electrochemical deposition to increase the electrode surface [141]. Signal amplification was also achieved via synergetic catalysis using DNAzyme-decorated AuPd nanoparticles. The signal was amplified by synergistic catalysis of G-quadruplex/hemin/HRP/AuPd/poly(o-phenylenediamine) bioconjugates, thus greatly enhancing the sensitivity of the biosensor [142]. Jeddi and Saiz presented the first approach to predict three-dimensional structures of aptamers from sequences by focusing explicitly on ssDNA hairpins [143]. Different 3D aptamer configurations resulted in different affinities, thus highlighting the role of 3D configuration of aptamers in their sensitivity, specificity, and reliability.

### 3.3. Aptamers as Therapeutics

Due to their ability to compete with small molecules and protein ligands and to inhibit their targets [144], aptamers are considered to be promising therapeutics. Furthermore, aptamers can activate the function of the target receptors or act as carriers for the delivery of therapeutic agents to the target cells or tissue [9]. For example, aptamers have the potential to act as antiviral agents. Previous work suggests that RNA aptamers against a synthetic derivative of gp120 can neutralize HIV-1 [145]. Furthermore, RNA aptamers against RIG-I can inhibit Newcastle disease virus (NDV), vesicular stomatitis virus (VSV), and influenza virus replication [144]. RNA aptamers able to efficiently inhibit protease and helicase activities were developed which target hepatitis C virus (HCV) NS3 helicase domain, thus inhibiting HCV [146,147,148]. Other aptamers in discovery and preclinical stages include those targeting cancer, such as those binding different growth factors (e.g. VEGF, bFGF, PDGF, KGF), NOX-A12 RNA aptamer binding the chemokine ligand CXCL12 [149] and NAS-24 DNA aptamer targeting vimentin involved in maintaining cell shape, cytoplasm integrity, and cytoskeleton stability [150]. Furthermore, aptamers in discovery and preclinical stages include those against bacterial infections, such as those caused by *E. coli*, *S. aureus*, *M. tuberculosis*, and *Salmonella* spp. [151,152]. A number of aptamers for immune disorders are in the preclinical stages too, including ssDNA aptamer BC007 targeting beta1-adrenoreceptor autoantibodies [153]. Furthermore, aptamers in the preclinical stages of development include those targeting neurodegenerative diseases, immune disorders, and inflammation [151].

A number of aptamers have entered clinical trials, such as for ocular diseases [154], haematologic diseases [155], and cancer [156]. Pegaptanib, a vascular endothelial growth factor (VEGF)-specific aptamer, was approved for therapeutic use for age-related macular degeneration. Pegaptanib blocks VEGF which plays a key role in pathological angiogenesis, for example, in ocular neovascular diseases, such as age-related macular degeneration (AMD) and diabetic macular oedema [157]. AS1411, a G-rich DNA aptamer against nucleolin, is undergoing phase II clinical trial for acute myeloid leukemia. The anti-tumor activity of AS1411 stems from its ability to bind cell surface nucleolin, thereby inhibiting DNA synthesis, preventing cell growth signaling, and inducing apoptosis [156]. AS1411 was shown to be efficient against a variety of cancer cells, including lung cancer [156], colorectal cancer [158], breast cancer [159], and hepatocellular carcinoma [160]. NOX-E36, l-RNA aptamer with 3′-PEG against chemokine (C-C motif) ligand 2, is in phase I clinical trial for Type 2 diabetes and diabetic nephropathy. NOX-E36 binds and neutralizes human chemokine CCL2 and related chemokines. This prevents infiltration of pro-inflammatory cells into the kidney and allows resolving of the existing inflammation over time [161,162]. Aptamers as therapeutics, including those in clinical trials, have been summarized in Table 3. The current list of the therapeutic aptamers in clinical trials which is being constantly updated can be found at the Clinical Trials website of the NIH U.S. National Library of Medicine (https://clinicaltrials.gov/) and in the recent review by Ismail and Alshaer [151].

Aptamers have been used successfully for the delivery of a variety of therapeutic reagents into the target cells and tissues [9]. Aptamer-based delivery systems include the aptamer-therapeutic oligonucleotide conjugates [163], aptamer-drug conjugates [164], and aptamer-decorated nanomaterials [9,165]. Examples of aptamers used for targeted drug delivery are shown in Table 4.

## 4. Conclusions and Future Directions

As described in this review, aptamers have a number of biotechnologically and medically-relevant applications; however, the main limitations for their in vivo therapeutic applications are target specific delivery. Cell-SELEX and whole animal in vivo SELEX can be employed to address these issues.

**Figure 4:**
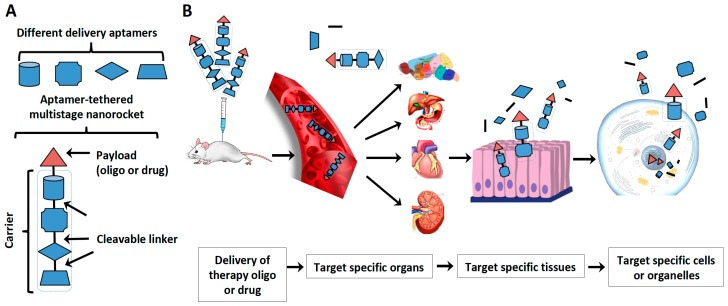
Aptamer-tethered multistage “nanorocket” for target-specific delivery. (**A**) An aptamer-tethered multistage “nanorocket” is a complex of two or more aptamers, each of which contains its own functional oligonucleotide. Serial stages of aptamers are mounted on top of another to build a carrier “nanorocket” for the target specific recognition and delivery. With appropriate linkages, each stage of aptamer “nanorocket” can freely rotate and perform its function. (**B**) The selected aptamers can be used in different stages of “nanorockets”, designed for tissue penetration, cell target recognition and cellular internalization. After reaching target, the recognition and delivering stages of the “nanorockets” can be cleaved and degraded by the cell leaving only the cargo oligos. Aptamers in multi-stage “nanorockets” can be used as tissue-, cell-type-, and cellular compartment-specific delivery systems.

Target site-specific delivery is a crucial problem in the therapeutic use of oligonucleotides, which can be overcome by employing aptamers in the form of a “nanorocket”. An aptamer-tethered multistage “nanorocket” is a complex of two or more aptamers, each of which contains its own functional oligonucleotide. Serial stages of aptamers are mounted on top of another to build a carrier “nanorocket” for payload drug, allowing delivery into selected tissues, cell types or even subcellular organelles (Figure 4A). To design and engineer the multistage “nanorocket”, whole animal-SELEX, tissue-SELEX, cell-SELEX and fractionation-SELEX can be used. This involves selection of the oligonucleotides pool for different targets. After incubation and isolation of the fraction of interest, such as specific animal tissues, cell types or subcellular fractions, those containing the binding complex (targets and oligonucleotide sequences) are partitioned from the unbound sequences and amplified by PCR. The process can be repeated until the pool is enriched for target-specific sequences. To increase the specificity, counter selection can be introduced.

Prior knowledge of the delivery mechanism, binding targets and delivery pathway through the tissue or cell surface barrier is unnecessary. Crucial are the types of tissues, cells and organelles used in the process, as they will be specifically recognized and targeted by the generated aptamers. The selected aptamers can be used in different stages of “nanorockets”, designed for tissue penetration, cell target recognition and cellular internalization. With appropriate linkages, each aptamer stage can freely rotate and perform its function. With cytosolic cleavable linkers, such as *N*-succinimidyl-4-(2-pyridyldithio) pentanoate (SPP) and *N*-succinimidyl-4-(2-pyridyldithio) butyrate (SPDB) or acid-cleavable hydrazone linkers for lysosome-specific release, the recognition and delivering stages of the “nanorockets” can be removed at the desired time point. After reaching the target, the recognition and delivering stages of the “nanorockets” can be cleaved and degraded by the cell leaving only the cargo oligos chemically modified to be resistant to the degradation. Modified aptamers in multi-stage “nanorockets” can be used as tissue-, cell-type-, and cellular compartment-specific delivery systems (Figure 4B).

**Figure 5:**
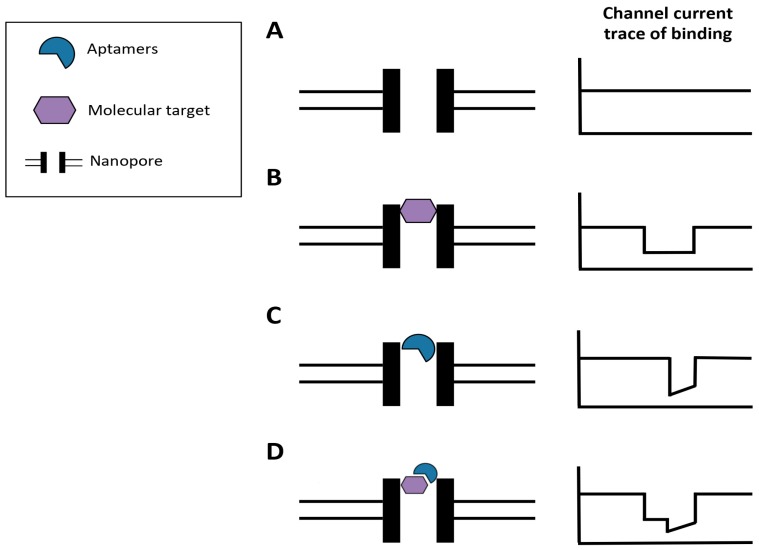
Flowchart of nanopore-based aptamer assaying. Nanopore sensors can be used to detect aptamer binding and selection in a real-time. Aptamer and target interaction can be detected by measuring the current disruption caused by molecules electrophoretically driven through the pore. (**A**) An ionic current is passed through the nanopore. The current changes as molecular target (**B**), aptamer (**C**), or aptamer–target complex (**D**) passes through the nanopore.

In term of diagnostics, a number of analytic devices were introduced to in the past years to improve performance and simplify the diagnostic procedure. AFM, microfluidic, and capillary electrophoresis were employed to couple with aptamers for high specific detection. AFM permits assessing of aptamer binding affinities during interaction by visualizing aptamer-target complexes and is considered a step forward towards real-time aptamer monitoring. In the future, a nanopore sensor (nano-scale pore with voltage applied across it) can be incorporated into the procedure, thus allowing real-time observation of binding. Single-molecule detection can be achieved by measuring the current disruption caused by molecules electrophoretically driven through the pore. The aptamer-protein target binding can be characterized by ion blockade level, and the binding evaluated in real-time (Figure 5).

Aptamers are cheap to manufacture, thermally stable and non-immunogenic. They specifically target harmful cells or tissues with minimal toxicity to the healthy ones, and can be chemically modified to facilitate their visualization, absorption, and delivery. Due to their specificity, stability, and versatility, aptamers embody the future of diagnostics and therapy.

## Figures and Tables

**Table 1:** Comparison between aptamers and antibodies. A comparison of critical features of aptamers shows how aptamers can supplement monoclonal antibodies.

	Aptamers	Antibodies
Stability	Withstand repeated rounds of denaturation/renaturation. Temperature resistant: stable at room temperature. Long shelf life (several years). Can be lyophilized. Degradable by nucleases. Resistant to proteases.	Easily denatured. Temperature sensitive and require refrigeration to avoid denaturation. Limited shelf life. Must be refrigerated for storage and transport. Degradable by proteases. Resistant to nucleases.
Synthesis	In vitro SELEX takes only 2–8 weeks. No batch-to-batch variation. Cheap to synthesize.	Produced in vivo. More than 6 months. Batch-to-batch variations. Laborious and expensive.
Target potential	From ions and small molecules to whole cells and live animals.	Targets must cause a strong immune response for antibodies to be produced.
Size	Small molecules.	Relatively large by comparison.
Modifiability	Aptamers can readily and easily be modified without affinity loss.	Modifications often lead to reduced activity.
Affinity	High and increased in multivalent aptamers.	Dependent on the number of epitopes on the antigen.
Specificity	Single point mutations identifiable.	Different antibodies might bind the same antigen.
Tissue uptake/kidney filtration	Fast.	Slow.

**Table 2:** Key aspects, advantages, and disadvantages of the currently used SELEX methods.

Method	Key Aspects	Advantages	Disadvantages
IP-SELEX	Includes immunoprecipitation.	Selects aptamers against proteins under normal physiological conditions. Increased affinity and specificity.	More time-consuming than standard SELEX.
Capture-SELEX	Oligonucleotide library is immobilized on a support instead of the targets to identify aptamers against small soluble molecules.	Suitable for the selection of aptamers against small molecules. Immobilization of the target not required. Used for the discovery of structure-switching aptamers.	Some oligonucleotides from the library might be not released/selected.
Cell-SELEX	Utilizes whole live cells as targets for selection of aptamers.	Prior knowledge of the target not required. Aptamers are selected against molecules in their native state. Many potential targets available on the cell surface. Protein purification not required.	Suitable for cell surface targets. Requires high level of technical expertise. Costly. Time consuming. Post SELEX identification of the target required.
CE-SELEX	Involves separation of ions based on electrophoretic mobility.	Fast. Only few (1–4) rounds of selection required. Reduced non-specific binding. Target immobilization not required.	Not suitable for small molecules. Expensive equipment.
M-SELEX	Combines SELEX with a microfluidic system.	Rapid. Very efficient (only small amounts of reagents needed). Applicable to small molecules. Automatable.	Low purity/recovery of aptamers. Target immobilization required.
AFM-SELEX	Employs AFM to create three-dimensional image of the sample surface.	Able to isolate high affinity aptamers. Fast (only 3–4 rounds required).	Expensive equipment required. Immobilization of target and aptamers required.
AEGIS-SELEX	Utilizes libraries with the artificially expanded genetic code.	High specificity of the selected aptamers.	Poor recognition of the unnatural bases by natural DNA polymerases.
Animal-SELEX	Aptamers are selected directly within live animal models.	Selected aptamers bind the targets in their natural environment. Prior knowledge of the target not required. Minimal optimization needed.	Time consuming (many rounds required).

**Table 3:** Aptamer as therapeutics.

Target	Aptamers	References
VEGF-165	SL (2)-B (DNA), RNV66 (DNA)	[166]
Nucleolin	FCL-II	[167]
CXCL12	NOX-A12	[168]
EGFR	TuTu2231, KDI130	[32]
Vimentin	NAS-24	[150]
E-selectin	ESTA	[169]
PD-1	MP7	[170]
CTLA-4	AptCTLA-4	[171]
C5a	AON-D21 I-aptamer	[172]
CD44/EpCAM	CD44-EpCAM aptamer	[173]
Thrombin	Anti-Thrombin aptamer	[174]

**Table 4:** Aptamers for the targeted drug delivery.

Target Name	Aptamer	Selection Target	Delivery/Application
Epidermal growth factor receptor (EGFR)	RNA	Purified extracellular domain of EGFR	Nanoparticle delivery
Immunoglobin heavy chain (IGHM)	DNA	Cell	Micelle nanoparticles for drug delivery
Mucin1 (MUC-1)	DNA	Recombinant peptides	Photodynamic therapy, radionuclide delivery
Prostate-specific membrane antigen (PSMA)	RNA	Purified extracellular domain of PSMA	siRNA delivery, Chemotherapeutic drug delivery
Protein tyrosine kinase-7 (PTK7)	DNA	Cell	Chemotherapeutic drug delivery

## References

[1] Strebhardt K., Ullrich A. (2008). Paul Ehrlich’s magic bullet concept: 100 years of progress. Nat. Rev. Cancer.

[2] Köhler G., Milstein C. (1975). Continuous cultures of fused cells secreting antibody of predefined specificity. Nature.

[3] Hudziak R.M., Lewis G.D., Winget M., Fendly B.M., Shepard H.M., Ullrich A. (1989). p185HER2 monoclonal antibody has antiproliferative effects in vitro and sensitizes human breast tumor cells to tumor necrosis factor. Mol. Cell. Biol..

[4] Di Gaetano N., Cittera E., Nota R., Vecchi A., Grieco V., Scanziani E., Botto M., Introna M., Golay J. (2003). Complement activation determines the therapeutic activity of rituximab in vivo. J. Immunol..

[5] Sandborn W.J., Feagan B.G., Rutgeerts P., Hanauer S., Colombel J.F., Sands B.E., Lukas M., Fedorak R.N., Lee S., Bressler B. (2013). Vedolizumab as induction and maintenance therapy for Crohn’s disease. N. Engl. J. Med..

[6] Sullenger B.A., Gallardo H.F., Ungers G.E., Gilboa E. (1991). Analysis of trans-acting response decoy RNA-mediated inhibition of human immunodeficiency virus type 1 transactivation. J. Virol..

[7] Ellington A.D., Szostak J.W. (1990). In vitro selection of RNA molecules that bind specific ligands. Nature.

[8] Tuerk C., Gold L. (1990). Systematic evolution of ligands by exponential enrichment: RNA ligands to bacteriophage T4 DNA polymerase. Science.

[9] Zhou J., Rossi J. (2017). Aptamers as targeted therapeutics: Current potential and challenges. Nat. Rev. Drug Discov..

[10] Bayat P., Nosrati R., Alibolandi M., Rafatpanah H., Abnous K., Khedri M., Ramezani M. (2018). SELEX methods on the road to protein targeting with nucleic acid aptamers. Biochimie.

[11] Kaur H. (2018). Recent developments in cell-SELEX technology for aptamer selection. Biochim. Biophys. Acta Gen. Subj..

[12] Zhuo Z., Yu Y., Wang M., Li J., Zhang Z., Liu J., Wu X., Lu A., Zhang G., Zhang B. (2017). Recent Advances in SELEX Technology and Aptamer Applications in Biomedicine. Int. J. Mol. Sci..

[13] Vorobyeva M.A., Davydova A.S., Vorobjev P.E., Venyaminova A.G. (2018). Key Aspects of Nucleic Acid Library Design for in Vitro Selection. Int. J. Mol. Sci..

[14] Randrianjatovo-Gbalou I., Rosario S., Sismeiro O., Varet H., Legendre R., Coppée J.Y., Huteau V., Pochet S., Delarue M. (2018). Enzymatic synthesis of random sequences of RNA and RNA analogues by DNA polymerase theta mutants for the generation of aptamer libraries. Nucleic Acids Res..

[15] Mayer G., Ahmed M.S., Dolf A., Endl E., Knolle P.A., Famulok M. (2010). Fluorescence-activated cell sorting for aptamer SELEX with cell mixtures. Nat. Protoc..

[16] Chang Y.C., Kao W.C., Wang W.Y., Yang R.B., Peck K. (2009). Identification and characterization of oligonucleotides that inhibit Toll-like receptor 2-associated immune responses. FASEB J..

[17] Wang C.W., Chung W.H., Cheng Y.F., Ying N.W., Peck K., Chen Y.T., Hung S.I. (2013). A new nucleic acid-based agent inhibits cytotoxic T lymphocyte-mediated immune disorders. J. Allergy Clin. Immunol..

[18] Mercier M.C., Dontenwill M., Choulier L. (2017). Selection of Nucleic Acid Aptamers Targeting Tumor Cell-Surface Protein Biomarkers. Cancers.

[19] Nutiu R., Li Y. (2005). In vitro selection of structure-switching signaling aptamers. Angew. Chem. Int. Ed. Engl..

[20] Lauridsen L.H., Doessing H.B., Long K.S., Nielsen A.T. (2018). A Capture-SELEX Strategy for Multiplexed Selection of RNA Aptamers Against Small Molecules. Methods Mol. Biol..

[21] Komarova N., Andrianova M., Glukhov S., Kuznetsov A. (2018). Selection, Characterization, and Application of ssDNA Aptamer against Furaneol. Molecules.

[22] Paniel N., Istamboulié G., Triki A., Lozano C., Barthelmebs L., Noguer T. (2017). Selection of DNA aptamers against penicillin G using Capture-SELEX for the development of an impedimetric sensor. Talanta.

[23] Reinemann C., Freiin von Fritsch U., Rudolph S., Strehlitz B. (2016). Generation and characterization of quinolone-specific DNA aptamers suitable for water monitoring. Biosens. Bioelectron..

[24] Nikolaus N., Strehlitz B. (2014). DNA-aptamers binding aminoglycoside antibiotics. Sensors.

[25] Spiga F.M., Maietta P., Guiducci C. (2015). More DNA-Aptamers for Small Drugs: A Capture-SELEX Coupled with Surface Plasmon Resonance and High-Throughput Sequencing. ACS Comb. Sci..

[26] Sefah K., Shangguan D., Xiong X., O’Donoghue M.B., Tan W. (2010). Development of DNA aptamers using Cell-SELEX. Nat. Protoc..

[27] Graham J.C., Zarbl H. (2012). Use of cell-SELEX to generate DNA aptamers as molecular probes of HPV-associated cervical cancer cells. PLoS ONE.

[28] Wang J., Gao T., Luo Y., Wang Z., Zhang Y., Pei R. (2019). In Vitro Selection of a DNA Aptamer by Cell-SELEX as a Molecular Probe for Cervical Cancer Recognition and Imaging. J. Mol. Evol..

[29] Hung L.Y., Wang C.H., Hsu K.F., Chou C.Y., Lee G.B. (2014). An on-chip Cell-SELEX process for automatic selection of high-affinity aptamers specific to different histologically classified ovarian cancer cells. Lab Chip.

[30] He J., Wang J., Zhang N., Shen L., Wang L., Xiao X., Wang Y., Bing T., Liu X., Li S. (2019). In vitro selection of DNA aptamers recognizing drug-resistant ovarian cancer by cell-SELEX. Talanta.

[31] Rong Y., Chen H., Zhou X.F., Yin C.Q., Wang B.C., Peng C.W., Liu S.P., Wang F.B. (2016). Identification of an aptamer through whole cell-SELEX for targeting high metastatic liver cancers. Oncotarget.

[32] Wang Y., Luo Y., Bing T., Chen Z., Lu M., Zhang N., Shangguan D., Gao X. (2014). DNA aptamer evolved by cell-SELEX for recognition of prostate cancer. PLoS ONE.

[33] Zhang K., Sefah K., Tang L., Zhao Z., Zhu G., Ye M., Sun W., Goodison S., Tan W. (2012). A novel aptamer developed for breast cancer cell internalization. ChemMedChem.

[34] Li W.M., Zhou L.L., Zheng M., Fang J. (2018). Selection of Metastatic Breast Cancer Cell-Specific Aptamers for the Capture of CTCs with a Metastatic Phenotype by Cell-SELEX. Mol. Ther. Nucleic Acids.

[35] Wu Q., Wang Y., Wang H., Wu L., Zhang H., Song Y., Zhu Z., Kang D., Yang C. (2018). DNA aptamers from whole-cell SELEX as new diagnostic agents against glioblastoma multiforme cells. Analyst.

[36] Maimaitiyiming Y., Yang C., Wang Y., Hussain L., Naranmandura H. (2019). Selection and characterization of novel DNA aptamer against colorectal carcinoma Caco-2 cells. Biotechnol. Appl. Biochem..

[37] Shi H., Cui W., He X., Guo Q., Wang K., Ye X., Tang J. (2013). Whole cell-SELEX aptamers for highly specific fluorescence molecular imaging of carcinomas in vivo. PLoS ONE.

[38] Thiel W.H., Thiel K.W., Flenker K.S., Bair T., Dupuy A.J., McNamara J.O., Miller F.J., Giangrande P.H. (2015). Cell-internalization SELEX: Method for identifying cell-internalizing RNA aptamers for delivering siRNAs to target cells. Methods Mol. Biol..

[39] Iaboni M., Fontanella R., Rienzo A., Capuozzo M., Nuzzo S., Santamaria G., Catuogno S., Condorelli G., de Franciscis V., Esposito C.L. (2016). Targeting Insulin Receptor with a Novel Internalizing Aptamer. Mol. Ther. Nucleic Acids.

[40] Zümrüt H.E., Batool S., Van N., George S., Bhandari S., Mallikaratchy P. (2017). Structural optimization of an aptamer generated from Ligand-Guided Selection (LIGS) resulted in high affinity variant toward mIgM expressed on Burkitt’s lymphoma cell lines. Biochim. Biophys. Acta Gen. Subj..

[41] Zumrut H.E., Ara M.N., Fraile M., Maio G., Mallikaratchy P. (2016). Ligand-Guided Selection of Target-Specific Aptamers: A Screening Technology for Identifying Specific Aptamers Against Cell-Surface Proteins. Nucleic Acid Ther..

[42] Zumrut H.E., Ara M.N., Maio G.E., Van N.A., Batool S., Mallikaratchy P.R. (2016). Ligand-guided selection of aptamers against T-cell Receptor-cluster of differentiation 3 (TCR-CD3) expressed on Jurkat.E6 cells. Anal. Biochem..

[43] Kim J.W., Kim E.Y., Kim S.Y., Byun S.K., Lee D., Oh K.J., Kim W.K., Han B.S., Chi S.W., Lee S.C. (2014). Identification of DNA aptamers toward epithelial cell adhesion molecule via cell-SELEX. Mol. Cells.

[44] Civit L., Taghdisi S.M., Jonczyk A., Haßel S.K., Gröber C., Blank M., Stunden H.J., Beyer M., Schultze J., Latz E. (2018). Systematic evaluation of cell-SELEX enriched aptamers binding to breast cancer cells. Biochimie.

[45] Mosing R.K., Bowser M.T. (2009). Isolating aptamers using capillary electrophoresis-SELEX (CE-SELEX). Methods Mol. Biol..

[46] Mendonsa S.D., Bowser M.T. (2005). In vitro selection of aptamers with affinity for neuropeptide Y using capillary electrophoresis. J. Am. Chem. Soc..

[47] Mendonsa S.D., Bowser M.T. (2004). In vitro evolution of functional DNA using capillary electrophoresis. J. Am. Chem. Soc..

[48] Dong L., Tan Q., Ye W., Liu D., Chen H., Hu H., Wen D., Liu Y., Cao Y., Kang J. (2015). Screening and Identifying a Novel ssDNA Aptamer against Alpha-fetoprotein Using CE-SELEX. Sci. Rep..

[49] Hamedani N.S., Müller J. (2016). Capillary Electrophoresis for the Selection of DNA Aptamers Recognizing Activated Protein C. Methods Mol. Biol..

[50] Luo Z., Zhou H., Jiang H., Ou H., Li X., Zhang L. (2015). Development of a fraction collection approach in capillary electrophoresis SELEX for aptamer selection. Analyst.

[51] Dong L., Zhou H., Zhao M., Gao X., Liu Y., Liu D., Guo W., Hu H., Xie Q., Fan J. (2018). Phosphorothioate-Modified AP613-1 Specifically Targets GPC3 when Used for Hepatocellular Carcinoma Cell Imaging. Mol. Ther. Nucleic Acids.

[52] Ahmad K.M., Oh S.S., Kim S., McClellen F.M., Xiao Y., Soh H.T. (2011). Probing the limits of aptamer affinity with a microfluidic SELEX platform. PLoS ONE.

[53] Leblebici P., Leirs K., Spasic D., Lammertyn J. (2019). Encoded particle microfluidic platform for rapid multiplexed screening and characterization of aptamers against influenza A nucleoprotein. Anal. Chim. Acta.

[54] Hung L.Y., Fu C.Y., Wang C.H., Chuang Y.J., Tsai Y.C., Lo Y.L., Hsu P.H., Chang H.Y., Shiesh S.C., Hsu K.F. (2018). Microfluidic platforms for rapid screening of cancer affinity reagents by using tissue samples. Biomicrofluidics.

[55] Sinha A., Gopinathan P., Chung Y.D., Lin H.Y., Li K.H., Ma H.P., Huang P.C., Shiesh S.C., Lee G.B. (2018). An integrated microfluidic platform to perform uninterrupted SELEX cycles to screen affinity reagents specific to cardiovascular biomarkers. Biosens. Bioelectron..

[56] Lou X., Qian J., Xiao Y., Viel L., Gerdon A.E., Lagally E.T., Atzberger P., Tarasow T.M., Heeger A.J., Soh H.T. (2009). Micromagnetic selection of aptamers in microfluidic channels. Proc. Natl. Acad. Sci. USA.

[57] Cho M., Xiao Y., Nie J., Stewart R., Csordas A.T., Oh S.S., Thomson J.A., Soh H.T. (2010). Quantitative selection of DNA aptamers through microfluidic selection and high-throughput sequencing. Proc. Natl. Acad. Sci. USA.

[58] Kim S., Kim Y., Kim P., Ha J., Kim K., Sohn M., Yoo J.S., Lee J., Kwon J.A., Lee K.N. (2006). Improved sensitivity and physical properties of sol-gel protein chips using large-scale material screening and selection. Anal. Chem..

[59] Bae H., Ren S., Kang J., Kim M., Jiang Y., Jin M.M., Min I.M., Kim S. (2013). Sol-gel SELEX circumventing chemical conjugation of low molecular weight metabolites discovers aptamers selective to xanthine. Nucleic Acid Ther..

[60] Park J.W., Lee S.J., Ren S., Lee S., Kim S., Laurell T. (2016). Acousto-microfluidics for screening of ssDNA aptamer. Sci. Rep..

[61] Liu X., Li H., Jia W., Chen Z., Xu D. (2016). Selection of aptamers based on a protein microarray integrated with a microfluidic chip. Lab Chip.

[62] Miyachi Y., Shimizu N., Ogino C., Kondo A. (2010). Selection of DNA aptamers using atomic force microscopy. Nucleic Acids Res..

[63] Takenaka M., Okumura Y., Amino T., Miyachi Y., Ogino C., Kondo A. (2017). DNA-duplex linker for AFM-SELEX of DNA aptamer against human serum albumin. Bioorg. Med. Chem. Lett..

[64] Kimoto M., Yamashige R., Matsunaga K., Yokoyama S., Hirao I. (2013). Generation of high-affinity DNA aptamers using an expanded genetic alphabet. Nat. Biotechnol..

[65] Sefah K., Yang Z., Bradley K.M., Hoshika S., Jiménez E., Zhang L., Zhu G., Shanker S., Yu F., Turek D. (2014). In vitro selection with artificial expanded genetic information systems. Proc. Natl. Acad. Sci. USA.

[66] Biondi E., Lane J.D., Das D., Dasgupta S., Piccirilli J.A., Hoshika S., Bradley K.M., Krantz B.A., Benner S.A. (2016). Laboratory evolution of artificially expanded DNA gives redesignable aptamers that target the toxic form of anthrax protective antigen. Nucleic Acids Res..

[67] Chen T., Romesberg F.E. (2014). Directed polymerase evolution. FEBS Lett..

[68] Cheng C., Chen Y.H., Lennox K.A., Behlke M.A., Davidson B.L. (2013). In vivo SELEX for Identification of Brain-penetrating Aptamers. Mol. Ther. Nucleic Acids.

[69] Chen L., He W., Jiang H., Wu L., Xiong W., Li B., Zhou Z., Qian Y. (2019). In vivo SELEX of bone targeting aptamer in prostate cancer bone metastasis model. Int. J. Nanomed..

[70] Fernández G., Moraga A., Cuartero M.I., García-Culebras A., Peña-Martínez C., Pradillo J.M., Hernández-Jiménez M., Sacristán S., Ayuso M.I., Gonzalo-Gobernado R. (2018). TLR4-Binding DNA Aptamers Show a Protective Effect against Acute Stroke in Animal Models. Mol. Ther..

[71] Lecocq S., Spinella K., Dubois B., Lista S., Hampel H., Penner G. (2018). Aptamers as biomarkers for neurological disorders. Proof of concept in transgenic mice. PLoS ONE.

[72] Mai J., Li X., Zhang G., Huang Y., Xu R., Shen Q., Lokesh G.L., Thiviyanathan V., Chen L., Liu H. (2018). DNA Thioaptamer with Homing Specificity to Lymphoma Bone Marrow Involvement. Mol. Pharm..

[73] Ospina-Villa J.D., López-Camarillo C., Castañón-Sánchez C.A., Soto-Sánchez J., Ramírez-Moreno E., Marchat L.A. (2018). Advances on Aptamers against Protozoan Parasites. Genes.

[74] Bruno J.G., Carrillo M.P., Phillips T., Andrews C.J. (2010). A novel screening method for competitive FRET-aptamers applied to E. coli assay development. J. Fluoresc..

[75] Bruno J.G., Phillips T., Carrillo M.P., Crowell R. (2009). Plastic-adherent DNA aptamer-magnetic bead and quantum dot sandwich assay for Campylobacter detection. J. Fluoresc..

[76] Hamula C.L., Zhang H., Guan L.L., Li X.F., Le X.C. (2008). Selection of aptamers against live bacterial cells. Anal. Chem..

[77] Cao X., Li S., Chen L., Ding H., Xu H., Huang Y., Li J., Liu N., Cao W., Zhu Y. (2009). Combining use of a panel of ssDNA aptamers in the detection of Staphylococcus aureus. Nucleic Acids Res..

[78] Chen F., Zhou J., Luo F., Mohammed A.B., Zhang X.L. (2007). Aptamer from whole-bacterium SELEX as new therapeutic reagent against virulent Mycobacterium tuberculosis. Biochem. Biophys. Res. Commun..

[79] Duan N., Wu S., Chen X., Huang Y., Wang Z. (2012). Selection and Identification of a DNA Aptamer Targeted to Vibrio parahemolyticus. J. Agric. Food Chem..

[80] Dwivedi H.P., Smiley R.D., Jaykus L.A. (2010). Selection and characterization of DNA aptamers with binding selectivity to Campylobacter jejuni using whole-cell SELEX. Appl. Microbiol. Biotechnol..

[81] Labib M., Zamay A.S., Muharemagic D., Chechik A.V., Bell J.C., Berezovski M.V. (2012). Aptamer-based viability impedimetric sensor for viruses. Anal. Chem..

[82] Gopinath S.C., Hayashi K., Kumar P.K. (2012). Aptamer that binds to the gD protein of herpes simplex virus-1 and efficiently inhibits viral entry. J. Virol..

[83] Fukuda K., Vishinuvardhan D., Sekiya S., Kakiuchi N., Shimotohno K., Kumar P.K., Nishikawa S. (1997). Specific RNA aptamers to NS3 protease domain of hepatitis C virus. Nucleic Acids Symp. Ser..

[84] Kumar P.K., Machida K., Urvil P.T., Kakiuchi N., Vishnuvardhan D., Shimotohno K., Taira K., Nishikawa S. (1997). Isolation of RNA aptamers specific to the NS3 protein of hepatitis C virus from a pool of completely random RNA. Virology.

[85] Boiziau C., Dausse E., Yurchenko L., Toulmé J.J. (1999). DNA aptamers selected against the HIV-1 trans-activation-responsive RNA element form RNA-DNA kissing complexes. J. Biol. Chem..

[86] Gopinath S.C., Kawasaki K., Kumar P.K. (2005). Selection of RNA-aptamer against human influenza B virus. Nucleic Acids Symp. Ser..

[87] Jang K.J., Lee N.R., Yeo W.S., Jeong Y.J., Kim D.E. (2008). Isolation of inhibitory RNA aptamers against severe acute respiratory syndrome (SARS) coronavirus NTPase/Helicase. Biochem. Biophys. Res. Commun..

[88] Nagarkatti R., de Araujo F.F., Gupta C., Debrabant A. (2014). Aptamer based, non-PCR, non-serological detection of Chagas disease biomarkers in Trypanosoma cruzi infected mice. PLoS Negl. Trop. Dis..

[89] Guerra-Pérez N., Ramos E., García-Hernández M., Pinto C., Soto M., Martín M.E., González V.M. (2015). Molecular and Functional Characterization of ssDNA Aptamers that Specifically Bind Leishmania infantum PABP. PLoS ONE.

[90] Cheung Y.W., Dirkzwager R.M., Wong W.C., Cardoso J., D’Arc Neves Costa J., Tanner J.A. (2018). Aptamer-mediated Plasmodium-specific diagnosis of malaria. Biochimie.

[91] Tang M.S.L., Shiu S.C., Godonoga M., Cheung Y.W., Liang S., Dirkzwager R.M., Kinghorn A.B., Fraser L.A., Heddle J.G., Tanner J.A. (2018). An aptamer-enabled DNA nanobox for protein sensing. Nanomedicine.

[92] Godonoga M., Lin T.Y., Oshima A., Sumitomo K., Tang M.S., Cheung Y.W., Kinghorn A.B., Dirkzwager R.M., Zhou C., Kuzuya A. (2016). A DNA aptamer recognising a malaria protein biomarker can function as part of a DNA origami assembly. Sci. Rep..

[93] Jain P., Chakma B., Singh N.K., Patra S., Goswami P. (2016). Aromatic Surfactant as Aggregating Agent for Aptamer-Gold Nanoparticle-Based Detection of Plasmodium Lactate Dehydrogenase. Mol. Biotechnol..

[94] Geldert A., Zhang X., Zhang H., Lim C.T. (2017). Enhancing the sensing specificity of a MoS. Analyst.

[95] Frith K.A., Fogel R., Goldring J.P.D., Krause R.G.E., Khati M., Hoppe H., Cromhout M.E., Jiwaji M., Limson J.L. (2018). Towards development of aptamers that specifically bind to lactate dehydrogenase of Plasmodium falciparum through epitopic targeting. Malar. J..

[96] Fraser L.A., Kinghorn A.B., Dirkzwager R.M., Liang S., Cheung Y.W., Lim B., Shiu S.C., Tang M.S.L., Andrew D., Manitta J. (2018). A portable microfluidic Aptamer-Tethered Enzyme Capture (APTEC) biosensor for malaria diagnosis. Biosens. Bioelectron..

[97] Dirkzwager R.M., Kinghorn A.B., Richards J.S., Tanner J.A. (2015). APTEC: Aptamer-tethered enzyme capture as a novel rapid diagnostic test for malaria. Chem. Commun..

[98] Choi S.J., Ban C. (2016). Crystal structure of a DNA aptamer bound to PvLDH elucidates novel single-stranded DNA structural elements for folding and recognition. Sci. Rep..

[99] Wang W.X., Cheung Y.W., Dirkzwager R.M., Wong W.C., Tanner J.A., Li H.W., Wu Y. (2017). Specific and sensitive detection of Plasmodium falciparum lactate dehydrogenase by DNA-scaffolded silver nanoclusters combined with an aptamer. Analyst.

[100] Iqbal A., Labib M., Muharemagic D., Sattar S., Dixon B.R., Berezovski M.V. (2015). Detection of Cryptosporidium parvum Oocysts on Fresh Produce Using DNA Aptamers. PLoS ONE.

[101] Ospina-Villa J.D., Dufour A., Weber C., Ramirez-Moreno E., Zamorano-Carrillo A., Guillen N., Lopez-Camarillo C., Marchat L.A. (2018). Targeting the polyadenylation factor EhCFIm25 with RNA aptamers controls survival in Entamoeba histolytica. Sci. Rep..

[102] Zheng C.Y., Pestilli F., Rokem A. (2014). Deconvolution of High Dimensional Mixtures via Boosting, with Application to Diffusion-Weighted MRI of Human Brain. Adv. Neural Inf. Process. Syst..

[103] Wang K., Fan D., Liu Y., Wang E. (2015). Highly sensitive and specific colorimetric detection of cancer cells via dual-aptamer target binding strategy. Biosens. Bioelectron..

[104] Ye X., Shi H., He X., Wang K., He D., Yan L., Xu F., Lei Y., Tang J., Yu Y. (2015). Iodide-Responsive Cu-Au Nanoparticle-Based Colorimetric Platform for Ultrasensitive Detection of Target Cancer Cells. Anal. Chem..

[105] Zhang F., Li S., Cao K., Wang P., Su Y., Zhu X., Wan Y. (2015). A Microfluidic Love-Wave Biosensing Device for PSA Detection Based on an Aptamer Beacon Probe. Sensors.

[106] Yang X., Zhuo Y., Zhu S., Luo Y., Feng Y., Xu Y. (2015). Selectively assaying CEA based on a creative strategy of gold nanoparticles enhancing silver nanoclusters’ fluorescence. Biosens. Bioelectron..

[107] Li X., An Y., Jin J., Zhu Z., Hao L., Liu L., Shi Y., Fan D., Ji T., Yang C.J. (2015). Evolution of DNA aptamers through in vitro metastatic-cell-based systematic evolution of ligands by exponential enrichment for metastatic cancer recognition and imaging. Anal. Chem..

[108] Wu X., Zhao Z., Bai H., Fu T., Yang C., Hu X., Liu Q., Champanhac C., Teng I.T., Ye M. (2015). DNA Aptamer Selected against Pancreatic Ductal Adenocarcinoma for in vivo Imaging and Clinical Tissue Recognition. Theranostics.

[109] Sun H., Tan W., Zu Y. (2016). Aptamers: Versatile molecular recognition probes for cancer detection. Analyst.

[110] Ababneh N., Alshaer W., Allozi O., Mahafzah A., El-Khateeb M., Hillaireau H., Noiray M., Fattal E., Ismail S. (2013). In vitro selection of modified RNA aptamers against CD44 cancer stem cell marker. Nucleic Acid Ther..

[111] Iwagawa T., Ohuchi S.P., Watanabe S., Nakamura Y. (2012). Selection of RNA aptamers against mouse embryonic stem cells. Biochimie.

[112] Burke D.H., Hoffman D.C., Brown A., Hansen M., Pardi A., Gold L. (1997). RNA aptamers to the peptidyl transferase inhibitor chloramphenicol. Chem. Biol..

[113] Kim Y.J., Kim Y.S., Niazi J.H., Gu M.B. (2010). Electrochemical aptasensor for tetracycline detection. Bioprocess Biosyst. Eng..

[114] Cruz-Aguado J.A., Penner G. (2008). Determination of ochratoxin a with a DNA aptamer. J. Agric. Food Chem..

[115] Kim S.E., Su W., Cho M., Lee Y., Choe W.S. (2012). Harnessing aptamers for electrochemical detection of endotoxin. Anal. Biochem..

[116] Jo M., Ahn J.Y., Lee J., Lee S., Hong S.W., Yoo J.W., Kang J., Dua P., Lee D.K., Hong S. (2011). Development of single-stranded DNA aptamers for specific Bisphenol a detection. Oligonucleotides.

[117] Zeng G., Zhang C., Huang D., Lai C., Tang L., Zhou Y., Xu P., Wang H., Qin L., Cheng M. (2017). Practical and regenerable electrochemical aptasensor based on nanoporous gold and thymine-Hg. Biosens. Bioelectron..

[118] Li L., Li B., Qi Y., Jin Y. (2009). Label-free aptamer-based colorimetric detection of mercury ions in aqueous media using unmodified gold nanoparticles as colorimetric probe. Anal. Bioanal. Chem..

[119] Cui L., Wu J., Ju H. (2016). Label-free signal-on aptasensor for sensitive electrochemical detection of arsenite. Biosens. Bioelectron..

[120] Kim M., Um H.J., Bang S., Lee S.H., Oh S.J., Han J.H., Kim K.W., Min J., Kim Y.H. (2009). Arsenic removal from Vietnamese groundwater using the arsenic-binding DNA aptamer. Environ. Sci. Technol..

[121] Oroval M., Coll C., Bernardos A., Marcos M.D., Martínez-Máñez R., Shchukin D.G., Sancenón F. (2017). Selective Fluorogenic Sensing of As(III) Using Aptamer-Capped Nanomaterials. ACS Appl. Mater. Interfaces.

[122] Chen Z., Li L., Mu X., Zhao H., Guo L. (2011). Electrochemical aptasensor for detection of copper based on a reagentless signal-on architecture and amplification by gold nanoparticles. Talanta.

[123] Mishra G.K., Sharma V., Mishra R.K. (2018). Electrochemical Aptasensors for Food and Environmental Safeguarding: A Review. Biosensors.

[124] Eissa S., Zourob M. (2017). In vitro selection of DNA aptamers targeting β-lactoglobulin and their integration in graphene-based biosensor for the detection of milk allergen. Biosens. Bioelectron..

[125] Hu W., Chen Q., Li H., Ouyang Q., Zhao J. (2016). Fabricating a novel label-free aptasensor for acetamiprid by fluorescence resonance energy transfer between NH2-NaYF4: Yb, Ho@SiO2 and Au nanoparticles. Biosens. Bioelectron..

[126] Madianos L., Tsekenis G., Skotadis E., Patsiouras L., Tsoukalas D. (2018). A highly sensitive impedimetric aptasensor for the selective detection of acetamiprid and atrazine based on microwires formed by platinum nanoparticles. Biosens. Bioelectron..

[127] Xu G., Huo D., Hou C., Zhao Y., Bao J., Yang M., Fa H. (2018). A regenerative and selective electrochemical aptasensor based on copper oxide nanoflowers-single walled carbon nanotubes nanocomposite for chlorpyrifos detection. Talanta.

[128] Sinha J., Reyes S.J., Gallivan J.P. (2010). Reprogramming bacteria to seek and destroy an herbicide. Nat. Chem. Biol..

[129] Fan L., Zhao G., Shi H., Liu M., Li Z. (2013). A highly selective electrochemical impedance spectroscopy-based aptasensor for sensitive detection of acetamiprid. Biosens. Bioelectron..

[130] Abraham K.M., Roueinfar M., Ponce A.T., Lussier M.E., Benson D.B., Hong K.L. (2018). In Vitro Selection and Characterization of a Single-Stranded DNA Aptamer Against the Herbicide Atrazine. ACS Omega.

[131] Wang K.Y., Krawczyk S.H., Bischofberger N., Swaminathan S., Bolton P.H. (1993). The tertiary structure of a DNA aptamer which binds to and inhibits thrombin determines activity. Biochemistry.

[132] Zhang W., Liu Q.X., Guo Z.H., Lin J.S. (2018). Practical Application of Aptamer-Based Biosensors in Detection of Low Molecular Weight Pollutants in Water Sources. Molecules.

[133] Zhou W., Huang P.J., Ding J., Liu J. (2014). Aptamer-based biosensors for biomedical diagnostics. Analyst.

[134] Hong L., Zhou F., Shi D., Zhang X., Wang G. (2017). Portable aptamer biosensor of platelet-derived growth factor-BB using a personal glucose meter with triply amplified. Biosens. Bioelectron..

[135] Wiedman G.R., Zhao Y., Mustaev A., Ping J., Vishnubhotla R., Johnson A.T.C., Perlin D.S. (2017). An Aptamer-Based Biosensor for the Azole Class of Antifungal Drugs. mSphere.

[136] Yang Y., Yang X., Zou X., Wu S., Wan D., Cao A., Liao L., Yuan Q., Duan X. (2017). Ultrafine Graphene Nanomesh with Large On/Off Ratio for High-Performance Flexible Biosensors. Adv. Funct. Mater..

[137] Chen M.-L., Chen J.-H., Ding L., Xu Z., Wen L., Wang L.-B., Cheng Y.-H. (2017). Study of the detection of bisphenol A based on a nano-sized metal–organic framework crystal and an aptamer. Anal. Methods.

[138] Saidur M.R., Aziz A.R., Basirun W.J. (2017). Recent advances in DNA-based electrochemical biosensors for heavy metal ion detection: A review. Biosens. Bioelectron..

[139] Xu S., Feng X., Gao T., Wang R., Mao Y., Lin J., Yu X., Luo X. (2017). A novel dual-functional biosensor for fluorometric detection of inorganic pyrophosphate and pyrophosphatase activity based on globulin stabilized gold nanoclusters. Anal. Chim. Acta.

[140] Alhadrami H.A., Chinnappan R., Eissa S., Rahamn A.A., Zourob M. (2017). High affinity truncated DNA aptamers for the development of fluorescence based progesterone biosensors. Anal. Biochem..

[141] Arroyo-Currás N., Scida K., Ploense K.L., Kippin T.E., Plaxco K.W. (2017). High Surface Area Electrodes Generated via Electrochemical Roughening Improve the Signaling of Electrochemical Aptamer-Based Biosensors. Anal. Chem..

[142] Wang X., Sun D., Tong Y.A., Zhong Y., Chen Z. (2017). A voltammetric aptamer-based thrombin biosensor exploiting signal amplification via synergetic catalysis by DNAzyme and enzyme decorated AuPd nanoparticles on a poly(o-phenylenediamine) support. Microchim. Acta.

[143] Jeddi I., Saiz L. (2017). Three-dimensional modeling of single stranded DNA hairpins for aptamer-based biosensors. Sci. Rep..

[144] Hwang S.Y., Sun H.Y., Lee K.H., Oh B.H., Cha Y.J., Kim B.H., Yoo J.Y. (2012). 5′-Triphosphate-RNA-independent activation of RIG-I via RNA aptamer with enhanced antiviral activity. Nucleic Acids Res..

[145] Mufhandu H.T., Gray E.S., Madiga M.C., Tumba N., Alexandre K.B., Khoza T., Wibmer C.K., Moore P.L., Morris L., Khati M. (2012). UCLA1, a Synthetic Derivative of a gp120 RNA Aptamer, Inhibits Entry of Human Immunodeficiency Virus Type 1 Subtype C. J. Virol..

[146] Nishikawa F., Kakiuchi N., Funaji K., Fukuda K., Sekiya S., Nishikawa S. (2003). Inhibition of HCV NS3 protease by RNA aptamers in cells. Nucleic Acids Res..

[147] Umehara T., Fukuda K., Nishikawa F., Sekiya S., Kohara M., Hasegawa T., Nishikawa S. (2004). Designing and analysis of a potent bi-functional aptamers that inhibit protease and helicase activities of HCV NS3. Nucleic Acids Symp. Ser..

[148] Urvil P.T., Kakiuchi N., Zhou D.M., Shimotohno K., Kumar P.K., Nishikawa S. (1997). Selection of RNA aptamers that bind specifically to the NS3 protease of hepatitis C virus. Eur. J. Biochem..

[149] Hoellenriegel J., Zboralski D., Maasch C., Rosin N.Y., Wierda W.G., Keating M.J., Kruschinski A., Burger J.A. (2014). The Spiegelmer NOX-A12, a novel CXCL12 inhibitor, interferes with chronic lymphocytic leukemia cell motility and causes chemosensitization. Blood.

[150] Zamay T.N., Kolovskaya O.S., Glazyrin Y.E., Zamay G.S., Kuznetsova S.A., Spivak E.A., Wehbe M., Savitskaya A.G., Zubkova O.A., Kadkina A. (2014). DNA-aptamer targeting vimentin for tumor therapy in vivo. Nucleic Acid Ther..

[151] Ismail S.I., Alshaer W. (2018). Therapeutic aptamers in discovery, preclinical and clinical stages. Adv. Drug Deliv. Rev..

[152] Pan Q., Yan J., Liu Q., Yuan C., Zhang X.L. (2017). A single-stranded DNA aptamer against mannose-capped lipoarabinomannan enhances anti-tuberculosis activity of macrophages through downregulation of lipid-sensing nuclear receptor peroxisome proliferator-activated receptor γ expression. Microbiol. Immunol..

[153] Wallukat G., Müller J., Haberland A., Berg S., Schulz A., Freyse E.J., Vetter R., Salzsieder E., Kreutz R., Schimke I. (2016). Aptamer BC007 for neutralization of pathogenic autoantibodies directed against G-protein coupled receptors: A vision of future treatment of patients with cardiomyopathies and positivity for those autoantibodies. Atherosclerosis.

[154] Gragoudas E.S., Adamis A.P., Cunningham E.T., Feinsod M., Guyer D.R. (2004). VEGF Inhibition Study in Ocular Neovascularization Clinical Trial Group. Pegaptanib for neovascular age-related macular degeneration. N. Engl. J. Med..

[155] Rusconi C.P., Scardino E., Layzer J., Pitoc G.A., Ortel T.L., Monroe D., Sullenger B.A. (2002). RNA aptamers as reversible antagonists of coagulation factor IXa. Nature.

[156] Bates P.J., Laber D.A., Miller D.M., Thomas S.D., Trent J.O. (2009). Discovery and development of the G-rich oligonucleotide AS1411 as a novel treatment for cancer. Exp. Mol. Pathol..

[157] Ng E.W., Shima D.T., Calias P., Cunningham E.T., Guyer D.R., Adamis A.P. (2006). Pegaptanib, a targeted anti-VEGF aptamer for ocular vascular disease. Nat. Rev. Drug Discov..

[158] Alibolandi M., Taghdisi S.M., Ramezani P., Hosseini Shamili F., Farzad S.A., Abnous K., Ramezani M. (2017). Smart AS1411-aptamer conjugated pegylated PAMAM dendrimer for the superior delivery of camptothecin to colon adenocarcinoma in vitro and in vivo. Int. J. Pharm..

[159] Wang Y., Chen X., Tian B., Liu J., Yang L., Zeng L., Chen T., Hong A., Wang X. (2017). Nucleolin-targeted Extracellular Vesicles as a Versatile Platform for Biologics Delivery to Breast Cancer. Theranostics.

[160] Cho Y., Lee Y.B., Lee J.H., Lee D.H., Cho E.J., Yu S.J., Kim Y.J., Kim J.I., Im J.H., Oh E.J. (2016). Modified AS1411 Aptamer Suppresses Hepatocellular Carcinoma by Up-Regulating Galectin-14. PLoS ONE.

[161] Kulkarni O., Pawar R.D., Purschke W., Eulberg D., Selve N., Buchner K., Ninichuk V., Segerer S., Vielhauer V., Klussmann S. (2007). Spiegelmer inhibition of CCL2/MCP-1 ameliorates lupus nephritis in MRL-(Fas)lpr mice. J. Am. Soc. Nephrol..

[162] Kulkarni O., Eulberg D., Selve N., Zöllner S., Allam R., Pawar R.D., Pfeiffer S., Segerer S., Klussmann S., Anders H.J. (2009). Anti-Ccl2 Spiegelmer permits 75% dose reduction of cyclophosphamide to control diffuse proliferative lupus nephritis and pneumonitis in MRL-Fas(lpr) mice. J. Pharmacol. Exp. Ther..

[163] Shu D., Li H., Shu Y., Xiong G., Carson W.E., Haque F., Xu R., Guo P. (2015). Systemic Delivery of Anti-miRNA for Suppression of Triple Negative Breast Cancer Utilizing RNA Nanotechnology. ACS Nano.

[164] Gijs M., Aerts A., Impens N., Baatout S., Luxen A. (2016). Aptamers as radiopharmaceuticals for nuclear imaging and therapy. Nucl. Med. Biol..

[165] Liang C., Guo B., Wu H., Shao N., Li D., Liu J., Dang L., Wang C., Li H., Li S. (2015). Aptamer-functionalized lipid nanoparticles targeting osteoblasts as a novel RNA interference-based bone anabolic strategy. Nat. Med..

[166] Gantenbein A.R., Sarikaya H., Riederer F., Goadsby P.J. (2015). Postoperative hemicrania continua-like headache—A case series. J. Headache Pain.

[167] Fan X., Sun L., Li K., Yang X., Cai B., Zhang Y., Zhu Y., Ma Y., Guan Z., Wu Y. (2017). The Bioactivity of d-/l-Isonucleoside- and 2′-Deoxyinosine-Incorporated Aptamer AS1411s Including DNA Replication/MicroRNA Expression. Mol. Ther. Nucleic Acids.

[168] Zboralski D., Hoehlig K., Eulberg D., Frömming A., Vater A. (2017). Increasing Tumor-Infiltrating T Cells through Inhibition of CXCL12 with NOX-A12 Synergizes with PD-1 Blockade. Cancer Immunol. Res..

[169] Morita Y., Kamal M., Kang S.A., Zhang R., Lokesh G.L., Thiviyanathan V., Hasan N., Woo S., Zhao D., Leslie M. (2016). E-selectin Targeting PEGylated-thioaptamer Prevents Breast Cancer Metastases. Mol. Ther. Nucleic Acids.

[170] Prodeus A., Abdul-Wahid A., Fischer N.W., Huang E.H., Cydzik M., Gariépy J. (2015). Targeting the PD-1/PD-L1 Immune Evasion Axis With DNA Aptamers as a Novel Therapeutic Strategy for the Treatment of Disseminated Cancers. Mol. Ther. Nucleic Acids.

[171] Huang B.T., Lai W.Y., Chang Y.C., Wang J.W., Yeh S.D., Lin E.P., Yang P.C. (2017). A CTLA-4 Antagonizing DNA Aptamer with Antitumor Effect. Mol. Ther. Nucleic Acids.

[172] Ajona D., Ortiz-Espinosa S., Moreno H., Lozano T., Pajares M.J., Agorreta J., Bértolo C., Lasarte J.J., Vicent S., Hoehlig K. (2017). A Combined PD-1/C5a Blockade Synergistically Protects against Lung Cancer Growth and Metastasis. Cancer Discov..

[173] Zheng J., Zhao S., Yu X., Huang S., Liu H.Y. (2017). Simultaneous targeting of CD44 and EpCAM with a bispecific aptamer effectively inhibits intraperitoneal ovarian cancer growth. Theranostics.

[174] Waters B., Lillicrap D. (2009). The molecular mechanisms of immunomodulation and tolerance induction to factor VIII. J. Thromb. Haemost..

